# Unified-Hydrophilic-Interaction/Anion-Exchange Liquid Chromatography Mass Spectrometry (Unified-HILIC/AEX/MS): A Single-Run Method for Comprehensive and Simultaneous Analysis of Polar Metabolome

**DOI:** 10.1021/acs.analchem.2c03986

**Published:** 2022-11-25

**Authors:** Kohta Nakatani, Yoshihiro Izumi, Masatomo Takahashi, Takeshi Bamba

**Affiliations:** † Division of Metabolomics/Mass Spectrometry Center, Medical Research Center for High Depth Omics, Medical Institute of Bioregulation, Kyushu University, 3-1-1 Maidashi, Higashi-ku, Fukuoka 812-8582, Japan; ‡ Department of Systems Life Sciences, Graduate School of Systems Life Sciences, Kyushu University, 3-1-1 Maidashi, Higashi-ku, Fukuoka 812-8582, Japan

## Abstract

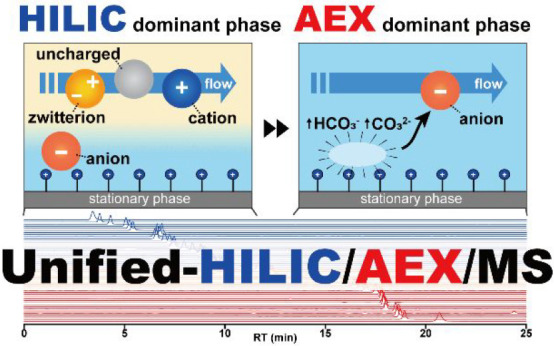

One of the technical challenges in the field of metabolomics is the development of a single-run method to detect the full complement of polar metabolites in biological samples. However, an ideal method to meet this demand has not yet been developed. Herein, we proposed a simple methodology that enables the comprehensive and simultaneous analysis of polar metabolites using unified-hydrophilic-interaction/anion-exchange liquid chromatography mass spectrometry (unified-HILIC/AEX/MS) with a polymer-based mixed amines column composed of methacrylate-based polymer particles with primary, secondary, tertiary, and quaternary amines as functional groups. The optimized unified-HILIC/AEX/MS method is composed of two consecutive chromatographic separations, HILIC-dominant separation for cationic, uncharged, and zwitterionic polar metabolites [retention times (RTs) = 0–12.8 min] and AEX-dominant separation for polar anionic metabolites (RTs = 12.8–26.5 min), by varying the ratio of acetonitrile to 40 mM ammonium bicarbonate solution (pH 9.8). A total of 400 polar metabolites were analyzed simultaneously through a combination of highly efficient separation using unified-HILIC/AEX and remarkably sensitive detection using multiple reaction monitoring-based triple quadrupole mass spectrometry (unified-HILIC/AEX/MS/MS). A nontargeted metabolomic approach using unified-HILIC/AEX high-resolution mass spectrometry (unified-HILIC/AEX/HRMS) also provided more comprehensive information on polar metabolites (3242 metabolic features) in HeLa cell extracts than the conventional HILIC/HRMS method (2068 metabolic features). Our established unified-HILIC/AEX/MS/MS and unified-HILIC/AEX/HRMS methods have several advantages over conventional techniques, including polar metabolome coverage, throughput, and accurate quantitative performance, and represent potentially useful tools for in-depth studies on metabolism and biomarker discovery.

## Introduction

Metabolomics is a comprehensive study of low-molecular-weight metabolites in biological samples.^1^ Almost all intermediates in metabolic pathways essential for maintaining biological activities, such as glycolysis, the pentose phosphate pathway, the tricarboxylic acid cycle, and amino acid and nucleic acid metabolism, are polar and ionic compounds (e.g., amino acids, nucleic acid constituents, organic acids, and coenzymes). Therefore, metabolite profiling of these polar metabolites may provide valuable insights into understanding metabolic activity and regulation.^2^ Recently, the rapid development of instrumentation for liquid chromatography mass spectrometry (LC/MS) and data mining techniques have contributed to detecting and identifying polar metabolites.^3,4^ However, comprehensive measurements of polar metabolites are still difficult owing to the various physicochemical properties of polar metabolites, such as polarity and charge status.^5^ The development of an ideal single-run method with comprehensive and rapid performance for polar metabolites will accelerate large-scale metabolomics studies (cohort studies using blood, urine, saliva, etc.) that require analysis of multiple samples. The single-run metabolomic approach is also useful for the analysis of trace samples (e.g., human clinical specimens), where available materials are limited. The features of the representative polar metabolomic methodology using LC/MS are summarized in Supporting Information, Table S-1.

Typical reversed-phase LC (RP-LC) is a widely used separation technique for hydrophobic metabolites based on the hydrophobic interaction between nonpolar side chains of C18 particles and hydrophobic moieties of metabolites.^6^ However, many polar and ionic compounds cannot be retained in the commonly used RP-LC columns because of the principle of hydrophobic interaction, limiting the polar metabolome information obtained by single-run RP-LC/MS analysis. To solve this problem, ion-pair (IP) RP-LC/MS (IP-RP-LC/MS) has been developed. Anionic polar metabolites, such as sugar phosphates, nucleotides, and organic acids, are poorly retained under typical RP-LC conditions; however, when deprotonated, these analytes form ionic interactions with oppositely charged IP reagents. For example, tributylamine (TBA) contains hydrophobic moieties that improve retention.^7^ Based on the same principle, analysis of cationic and zwitterionic polar metabolites (e.g., bases, nucleosides, and amino acids) can be performed by IP-RP-LC/MS using anionic IP reagents, such as heptafluorobutyric acid (HFBA).^8^ However, because high-concentration IP reagents contaminate the LC/MS equipment, the effects of IP reagents on LC/MS equipment cannot be ignored. In general, it is essential to use dedicated LC/MS instruments that use TBA or HFBA reagents.^4^

Mixed-mode RP-LC columns in which ion-exchange ligands are blended, mixed, or embedded within alkyl functional groups have recently been developed as an alternative strategy for increasing the coverage of observable polar metabolites. For example, a Scherzo SM-C18 mixed-mode RP-LC column retained polar metabolites better than typical C18 columns.^9^ However, mixed-mode RP-LC/MS techniques are potentially limited by the strong ion-exchange interactions between the stationary phase and physiologically important polar metabolites (coenzyme A, acetyl-CoA, ATP, etc.), for which elution may require increased levels of salt in the mobile phase that are not dissolved in organic solvents required for RP-LC separation.^4^

Advances in suppressor technology have enabled the coupling of an ion chromatograph with a mass spectrometer; ion chromatography (IC) with an anion-exchange (AEX) column coupled with mass spectrometry (IC/MS) is recognized as a robust analytical method for anionic polar metabolites.^10,11^ However, IC/MS, similar to IP-RP-LC/MS, cannot, in principle, separate and detect both cationic and anionic polar metabolites simultaneously.

Hydrophilic interaction chromatography (HILIC) is a separation technique in which polar compounds are retained by partitioning of the analyte, which occurs between the low-polarity organic mobile phase and the hydrated water layer on the surface of the polar stationary phase.^12^ Thus, HILIC can separate compounds according to their hydrophilicity. Furthermore, in recent years, new HILIC column stationary phases have been developed, in which ionic interactions and hydrogen bonding work together with partition phenomena to improve the coverage of measurable polar compounds.^13^ For example, aminopropyl silica columns with positively charged stationary phase surfaces are effective for the analysis of polar metabolites because the HILIC and AEX modes work simultaneously.^14^ However, aminopropyl silica columns exhibit poor stability owing to column bleeding by self-decomposition or irreversible adsorption of carbohydrates; hence, the use of aminopropyl silica columns has decreased in recent years.^15^ Another valid HILIC column, the zwitterionic column (e.g., ZIC-HILIC column or iHILIC Fusion (P) column), has both positive and negative charge sites embedded in silica or polymer particles, making it suitable for HILIC/MS analysis of a variety of polar metabolites.^16−18^ However, the ZIC-HILIC/MS method does not provide a highly sensitive and comprehensive analysis while maintaining good peak shape (baseline peak width < 1.0 min) and retention (retention factor, *k* ≥ 2) of all cationic and anionic polar metabolites.^19^ Thus, at present, a combination of several measurement methods is the only way to achieve a reasonably comprehensive analysis of the polar metabolites in real samples.^20^

The development of large compound databases (e.g., Pubchem^21^) and physicochemical property prediction software (e.g., ChemAxon Calculators and Predictors; https://www.chemaxon.com/) has greatly contributed to the construction of chromatographic theory for metabolomics. For example, a few studies characterized the relationship or transition between hydrophilic partitioning and/or ionic adsorption mechanisms in HILIC using authentic standards with physicochemical property information (log *P*_ow_, p*K*_a_, p*K*_b_, charge characteristics, molecular distribution, etc.).^19,22^ Continuous efforts to achieve efficient chromatographic separation and sensitive MS detection based on the physicochemical properties of the mobile phase, stationary phase, and analyte will lead to new proposals for the comprehensive and simultaneous measurement of the polar metabolome.

The objective of the present study was to develop a single-run method to comprehensively analyze polar metabolites. To develop this ideal method, the effects of LC conditions, including mobile phase solvents, types and concentrations of additives, and mobile phase pH, on the retention behavior and peak shape of 52 representative hydrophilic metabolites (amino acids, nucleic acid constituents, organic acids, and coenzymes) were evaluated using a polymer-based mixed amines column. Our detailed investigation, including simulations of molecular/ionic distributions of analytes, additives in the mobile phase, and functional groups in the stationary phase, successfully demonstrated the simultaneous analysis of polar metabolites with different charge characteristics using two consecutive chromatographic separations in HILIC and AEX modes and detection using a highly sensitive mass spectrometer. We termed this novel single-run analytical method “unified-hydrophilic-interaction/anion-exchange liquid chromatography mass spectrometry (unified-HILIC/AEX/MS)”. The established unified-HILIC/AEX/MS method has greater coverage of polar metabolites than conventional HILIC/MS methods, in both targeted and nontargeted metabolomics.

## Experimental Section

### Chemicals and Reagents

Phosphate-buffered saline (PBS), Dulbecco’s modified Eagle’s medium (DMEM) containing glucose (25 mM), Ham’s F-12 nutrient mixture, penicillin-streptomycin solution (10000 U/mL penicillin, 10000 μg/mL streptomycin), 10% (v/v) fetal bovine serum (FBS), and trypsin-EDTA solution (0.25% trypsin and 1 mM EDTA) were obtained from Thermo Fisher Scientific Inc. (Waltham, MA, U.S.A.). LC–MS-grade water, acetonitrile, and methanol were purchased from Kanto Chemical Co., Inc. (Tokyo, Japan). HPLC-grade chloroform and 28% (v/v) ammonium hydroxide were purchased from Nacalai Tesque Inc. (Kyoto, Japan). LC–MS-grade acetic acid and LC–MS-grade ammonium bicarbonate were purchased from Fujifilm Wako Pure Chemical Co. (Osaka, Japan). LC–MS-grade ammonium acetate was purchased from Merck (Darmstadt, Germany). Authentic standards were obtained from Nacalai Tesque, Inc., Fujifilm Wako Pure Chemical Co., and Merck. Stable isotope labeled authentic standards of ^18^O_9_-ATP (purity 92.9%), including ^18^O_6_-ADP (6.4%) and ^18^O_3_-AMP (0.7%), and ^13^C_2_, ^15^N-reduced glutathione (GSH) (purity 95.4%), including ^13^C_4_, ^15^N_2_-oxidized glutathione (GSSG; 4.6%; Supporting Information, Figure S-1) were obtained from Taiyo Nippon Sanso Co. Ltd. (Tokyo, Japan).

### Metabolites Extraction

Metabolite extraction for HeLa cells (American Type Culture Collection) was performed using the Bligh and Dyer method^23^ with some modifications. The details of the cell culturing and sample preparation methods for direct injection (DI) and centrifugal concentration/freeze-drying (CCFD) samples are described in the Supporting Information. A schematic diagram of the DI and CCFD operations is shown in Supporting Information, Figure S-2.

### Analytical Conditions for Targeted Metabolome Analysis

Targeted liquid chromatography triple quadrupole mass spectrometry (LC/MS/MS) analyses were performed using a Nexera X2 UHPLC system (Shimadzu Co., Kyoto, Japan) coupled with an LCMS-8060 triple quadrupole mass spectrometer (TQMS, Shimadzu Co.) with a heated electrospray ionization source. The LC system was equipped with two binary pumps (LC-30AD), an autosampler (SIL-30AC), and a temperature-controlled column oven (CTO-20AC). The pump and autosampler components were replaced with a Nexera wide pH kit, and the LC system was converted to a wide pH range (pH 1–14). The LC mobile phase conditions used to evaluate the separation behavior were as follows: mobile phase (A), 10, 20, or 40 mM ammonium bicarbonate (ABC) aqueous solutions, 10, 20, or 40 mM ammonium acetate (AA) aqueous solutions, or a mixture of equal volumes of 20 mM ABC solution and 20 mM AA solution; and mobile phase (B), acetonitrile. The pH (3.6, 7.0, or 9.8) of the aqueous mobile phase (A) was adjusted by adding acetic acid or 28% ammonium hydroxide, respectively. The three LC columns used to investigate the unified-HILIC/AEX separation mechanism were as follows: glycerol dimethacrylate-based bare polymer column (prototype), which was prepared by a radical polymerization of glycerol dimethacrylate, 3.6 μm particle-size, 2.1 mm i.d. × 150 mm (Showa Denko Materials Techno Service Co., Ltd., Ibaraki, Japan); spacer-modified (i.e., glycerol-modified) methacrylate polymer column (prototype), which was prepared from glycerol dimethacrylate-based bare polymer with epichlorohydrin (chloromethyloxirane) followed by hydrolysis, 3.6 μm particle-size, 2.1 mm i.d. × 150 mm (Showa Denko Materials Techno Service Co., Ltd.); and ammonium and amino-mixed spacer-modified methacrylate polymer column (mixed amines polymer column, GL-HilicAex, prototype), which was prepared by treating glycerol dimethacrylate bare polymer with epichlorohydrin followed by amination with polyethylenimine, 3.6 μm particle size, 2.1 mm i.d. × 150 mm (Showa Denko Materials Techno Service Co., Ltd.). The five LC columns used for chromatographic performance evaluation were as follows: Inertsil SIL-100A (bare-silica column), 3 μm particle size, 2.1 mm i.d. × 150 mm (GL Sciences Inc., Tokyo, Japan); Inertsil NH_2_ (amino-silica column), 3 μm particle size, 2.1 mm i.d. × 150 mm (GL Sciences Inc.); iHLIC-Fusion (P) (zwitterionic-polymer column), 5 μm particle size, 2.1 mm i.d. × 150 mm (HILICON, Umea, Sweden); ZIC-pHILIC (zwitterionic-polymer column), 5 μm particle size, 2.1 mm i.d. × 150 mm (Merck); and GL-HilicAex (mixed amines polymer column, prototype), 3.6 μm particle size, 2.1 mm i.d. × 150 mm (Showa Denko Materials Techno Service Co., Ltd.). The final LC analysis conditions were as follows: injection volume, 1 μL for standard solutions and 5 μL for HeLa cell extracts; flow rate, 0.4 mL/min; column temperature, 40 °C; mobile phase (A), 40 mM ABC aqueous solution at pH 9.8; and mobile phase (B), acetonitrile. The optimized gradient conditions were as follows: 95% B, 0–0.5 min; 95–40% B, 0.5–15.5 min; 40–0% B, 15.5–16.5 min; 0% B, 16.5–26.5 min; 0–95% B, 26.5–27.5 min; and 95% B, 27.5–35 min. The details of the multiple reaction monitoring (MRM) conditions are described in the Supporting Information. The optimized MRM parameters for the 400 polar metabolites are shown in Supporting Information, Table S-2. To create calibration curves for each polar metabolite, standard solutions were prepared at concentrations of 0, 1, 4, 10, 40, 100, 400, 1000, 4000, 10000, 40000, and 100000 nM. The LC/MRM data analysis was performed using LabSolutions, ver. 5.91 (Shimadzu Co.).

### Analytical Conditions for Nontargeted Metabolome Analysis

Nontargeted liquid chromatography high-resolution mass spectrometry (LC/HRMS) analyses were performed using a wide pH version Nexera X2 UHPLC system (Shimadzu Co.) coupled with a Q Exactive high-performance benchtop quadrupole Orbitrap mass spectrometer (Thermo Fisher Scientific Inc.) with a heated electrospray ionization source. LC analytical conditions were identical to those used in the targeted metabolome analysis method. The details of the full scanning HRMS analysis conditions are described in the Supporting Information. The Compound Discoverer ver. 3.0 (Thermo Fisher Scientific Inc.) was used for HRMS data processing.^24^ The details of the peak alignment and detection procedures are described in the Supporting Information.

### Calculation of the Structural Properties

Marvin was used for drawing chemical structures, and Calculator Plugins were used for structure property prediction and calculation (Marvin 17.29.0, 2017; ChemAxon Ltd., Budapest, Hungary; http://www.chemaxon.com). The physicochemical properties of 52 representative hydrophilic metabolites were extracted from the PubChem database^21^ or predicted using ChemAxon MarvinSketch (ChemAxon Ltd.; Supporting Information, Table S-3).

## Results and Discussion

### Optimization of MRM Conditions and Calculation of Chemical Properties of the Targeted Compounds

LC/MS/MS in MRM mode has attracted attention for widely targeted metabolome analysis owing to its selectivity, high sensitivity, and good quantitative performance.^25,26^ The MRM transitions (precursor ion, collision energy, product ion, and prequadrupole focusing voltages) of 400 polar metabolites were optimized by flow injection analysis of each authentic standard, with up to three MRM transitions for each metabolite (Supporting Information, Table S-2).

To investigate the retention behavior of hydrophilic metabolites by LC/MS/MS, 52 characteristic metabolites (amino acids, nucleic acid constituents, organic acids, coenzymes, etc.) were selected, and their chemical properties, including log *P*_ow_, strongest acidic p*K*_a_, second strongest p*K*_a_, strongest basic p*K*_b_, and second strongest p*K*_b_, are summarized in Supporting Information, Table S-3. Based on molecular/ion distribution at pH 7.0, we classified 52 representative polar metabolites into four groups: (i) net positively charged metabolites (cationic metabolites), (ii) uncharged metabolites, (iii) net neutral charged metabolites (zwitterionic metabolites), and (iv) net negatively charged metabolites (anionic metabolites; Supporting Information, Table S-3). Using the physicochemical properties of these 52 compounds, we examined the LC conditions and discussed their separation behavior.

### Column Design for Polar Metabolome Analysis

To achieve comprehensive single-run measurements of polar metabolites, it is necessary to develop column stationary phases that can interact simultaneously or stepwise with polar metabolites having different charge properties. Therefore, complex interactions such as ion exchange, in addition to interactions through HILIC distribution, as in the previous development of aminopropyl silica columns,^13^ will be required. In addition, the strength of the ionic interaction between the analyte and stationary phase of the column was determined based on the charge state of both the analyte and polar functional groups of the stationary phase. Because the charge state of the analyte and stationary phase are affected by the proton concentration of the mobile phase, the pH of the mobile phase is an important factor in determining ionic interactions. Thus, polymeric packing materials that can be used over a wide pH range are important for the development of new polar metabolomic methods. To propose a novel polar metabolome analysis method, we developed a mixed amines polymer column (GL-HilicAex) consisting of methacrylate-based polymer particles with primary, secondary, tertiary, and quaternary amines as functional groups that can be used over a wide pH range (pH 2–13).

### Optimization of LC Conditions Using a Mixed Amines Polymer Column

Because compounds adsorbed by ionic interactions could be eluted by the ion-exchange effect of the salts in the mobile phase, the type and concentration of the salts are also important factors for the elution of the adsorbed compound. We first evaluated the effect of the mobile phase additives and their concentrations on the chromatographic performance of the mixed amines polymer columns. The LC gradient conditions using the mixed amines polymer column were set to 95% acetonitrile, as in the initial conditions of HILIC, followed by a stepwise increase in the ratio of AA or ABC aqueous solutions, and finally replaced by 100% aqueous solution to elute the polar metabolites (see the Experimental Section for details). The retention times (RTs) of each polar compound acquired under each condition are shown in Supporting Information, Table S-4.

**Figure 1:**
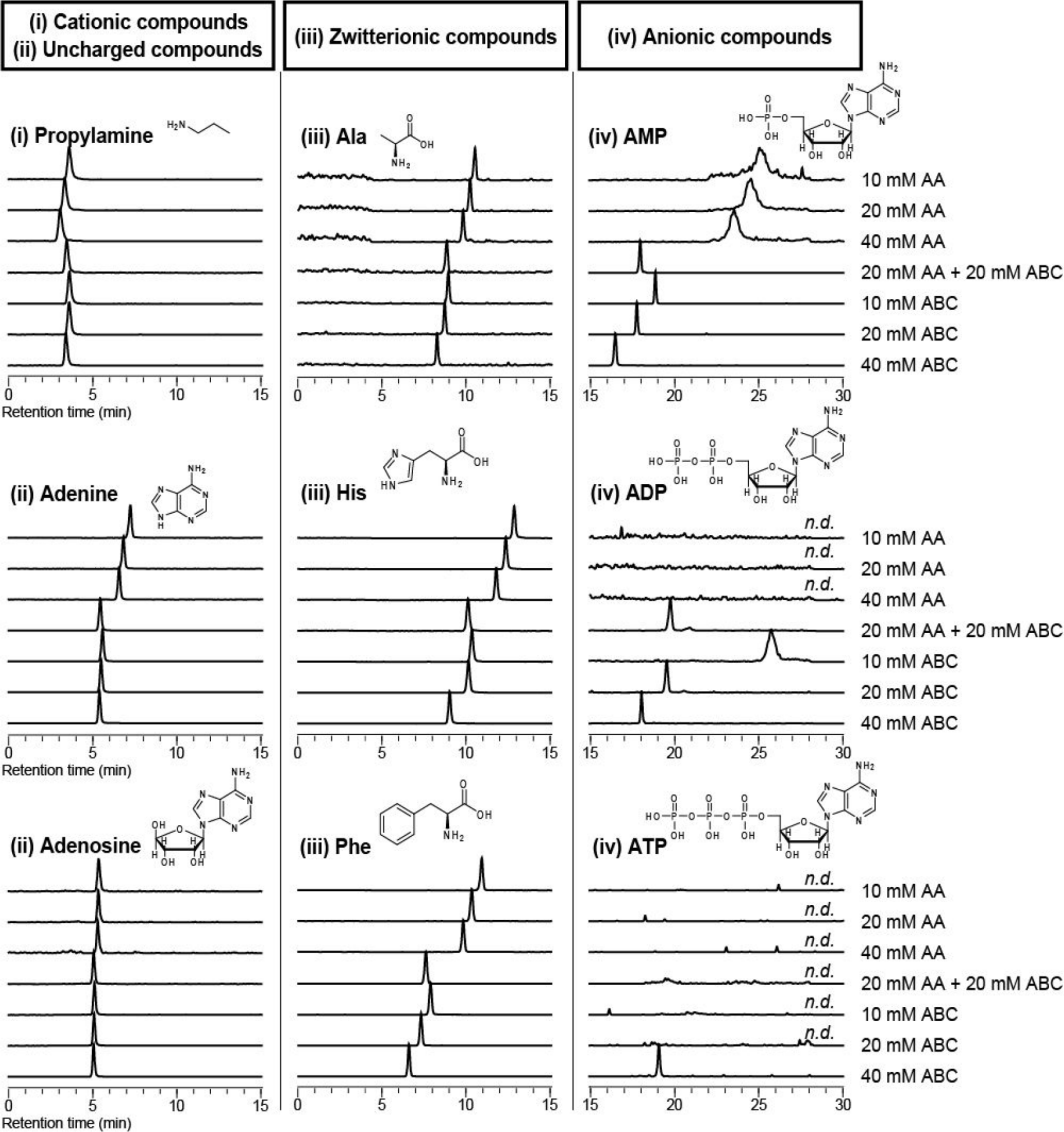
Effect of additive type and its concentration in the aqueous mobile phase (pH 9.8) on separation and elution of (i) cationic, (ii) uncharged, (iii) zwitterionic, and (iv) anionic polar metabolites by a mixed amines polymer column.

Polar metabolite standards of groups (iii) and (iv) eluted faster with increasing additive concentration at pH 9.8, and this tendency was more pronounced for the ABC solution than for the AA solution (Figure 1). Because the compounds in groups (iii) and (iv) have anionic moieties, such as carboxyl or phosphate groups, the zwitterionic/anionic compounds could be interacted/adsorbed to the positively charged stationary phase through ionic interactions. Therefore, the result of faster elution of groups (iii) and (iv) with increasing concentration of the additive suggests that the anions adsorbed on the ammonium and/or amino groups of the stationary phase were exchanged with eluent ions, such as CH_3_COO^–^, HCO_3_^–^, or CO_3_^2–^. Additionally, in IC, the larger the valence and radius of the ion, the stronger the adsorption to the ion-exchange group;^27^ this theory could be adapted to explain why ABC solutions elute zwitterionic/anionic metabolites faster than AA solutions. To discuss these results, the molecular/ionic distributions of the additive ions in the mobile phase were simulated using ChemAxon software. At pH 9.8, all AA-derived anions existed as monovalent anions (CH_3_COO^–^), while 87% of ABC-derived anions existed as monovalent anions (HCO_3_^–^) and 13% as divalent anions (CO_3_^2–^; Supporting Information, Figure S-3a). Thus, the effective action of the divalent anions (CO_3_^2–^) produced at pH 9.8 may be the reason why larger multivalent anions, such as ATP, were able to elute in the 40 mM ABC solution (Figure 1). In addition, the distributions of molecular/ionic states of the functional groups in the stationary phase were simulated (Supporting Information, Figure S-3b). The simulation results showed that 34, 16, and 63% of primary, secondary, and tertiary amino groups, respectively, were in the uncharged state at pH 9.8; however, quaternary ammonium groups were in the cationic state, regardless of pH. Thus, at pH 9.8, the net positive charge of the stationary phase decreases, and ionic interactions with anions become weaker, which may be one reason for the consequent successful elution of multivalent anionic metabolites, such as ATP.

**Figure 2:**
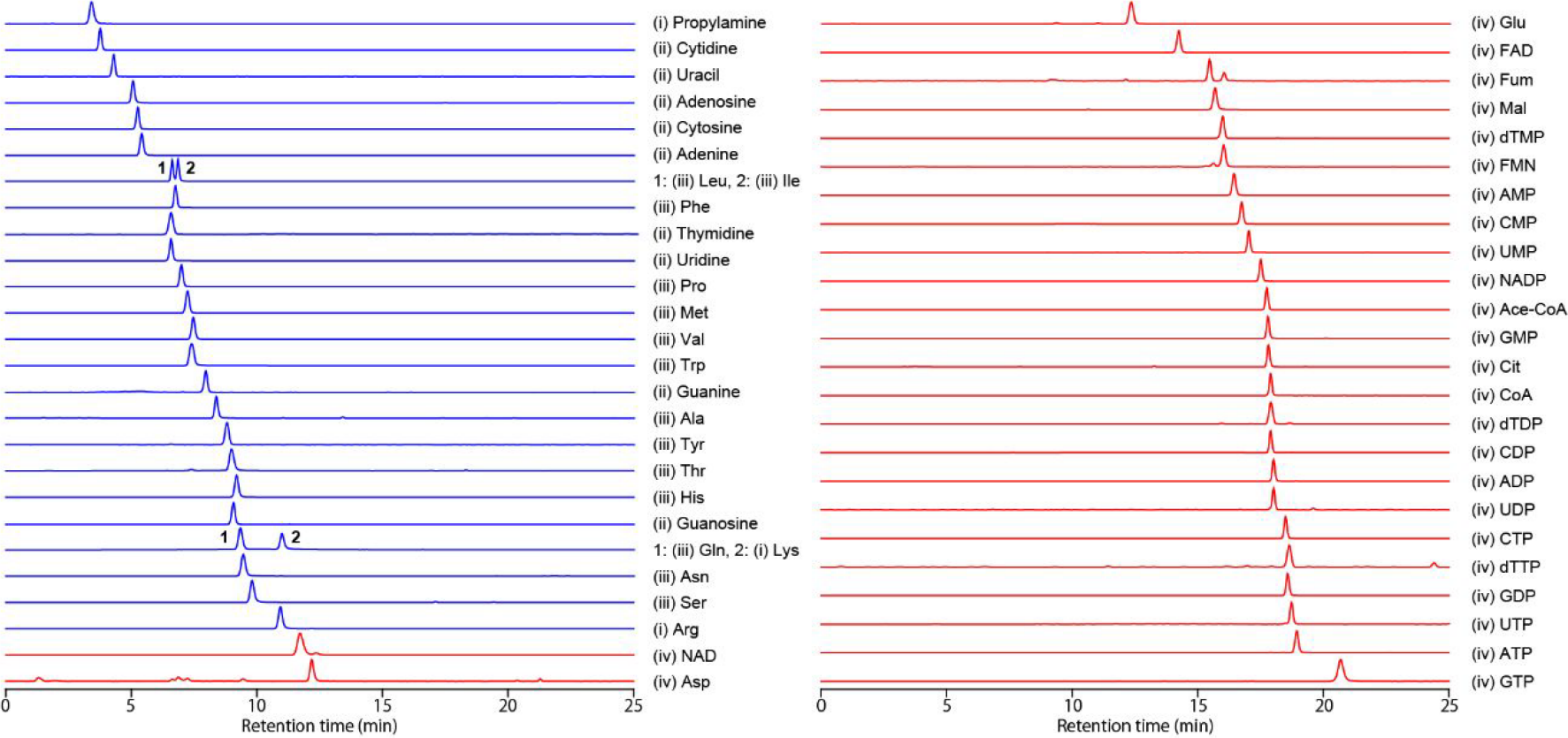
LC/MS/MS chromatograms of 52 representative polar metabolite standards in groups (i), (ii), (iii), and (iv) measured on a mixed amines polymer column. Mobile phase A, 40 mM ABC solution at pH 9.8; mobile phase B, acetonitrile. Other LC/MS/MS parameters are detailed in the Experimental Section.

Further, the retention behavior of several zwitterionic and anionic compounds was investigated at pH 3.6, 7.0, and 9.8 for the aqueous mobile phase (Supporting Information, Figure S-4). The retention of zwitterionic and anionic compounds was found to be stronger under acidic and neutral conditions than under basic conditions. For anionic metabolites, in particular, the effect of pH was significant, with sharp peaks eluting only under basic conditions. These experimental results were reasonable with simulations of the charge state of the additive and stationary phase. To summarize this section, simultaneous separation and detection of all 52 representative polar metabolite standards in groups (i), (ii), (iii), and (iv) was achieved by LC/MS/MS using a mixed amines polymer column by optimizing the type and concentration of additives and the pH of the aqueous mobile phase (i.e., 40 mM ABC solution at pH 9.8; Figure 2).

### Consideration of Separation Mechanisms: Characterization of Unified-HILIC/AEX Mode

**Figure 3:**
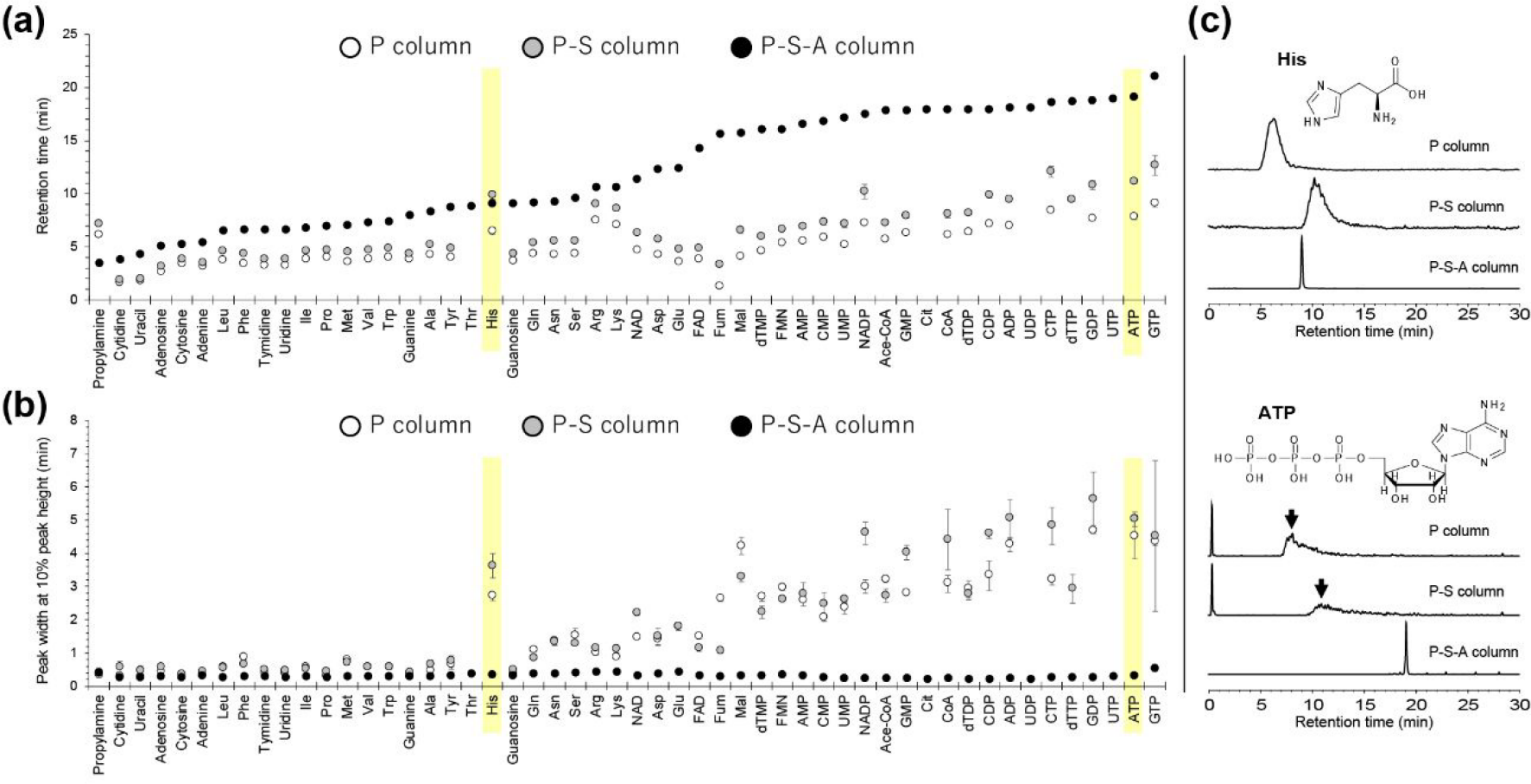
Comparison of RTs (a) and peak widths at 10% peak height (*W*_0.1_) (b) of 52 representative polar metabolites using a glycerol dimethacrylate-based bare polymer column (P column), a spacer-modified (i.e., glycerol-modified) methacrylate polymer column (P–S column), and a mixed amines polymer column (P–S–A column) under identical LC/MS/MS conditions. Values are presented as the mean ± standard deviation (*n* = 3). (c) Representative LC/MRM chromatograms of polar metabolites (His and ATP) under three different column conditions.

To further characterize the separation mechanism of the mixed amines polymer column (defined in this study as the P–S–A column or GL-HilicAex) under optimized LC/MS/MS conditions, we compared the retention behavior of 52 polar metabolites using glycerol dimethacrylate-based bare polymer (P column) and spacer-modified (i.e., glycerol-modified) methacrylate polymer columns (P–S column). The RTs and peak widths at 10% peak height (*W*_0.1_) obtained from LC/MS/MS using each of the three columns are shown in Figure 3a,b and Supporting Information, Table S-5. The LC/MRM chromatograms of histidine (His) and ATP are shown in Figure 3c as a comparative example using each of the three columns. The following three key observations were made in this experiment: first, 50 representative polar metabolites, except for propylamine and His, were strongly retained in the order P–S–A column > P–S column > P column (Figure 3a); second, the difference in RTs between the P–S–A column and the P–S or P columns was particularly large for group (iv) anionic polar metabolites (Figure 3a); third, weakly retained polar compounds, that is, metabolites with RTs < 12.8 min in LC/MS/MS using the P–S–A column, tended to elute with good peaks with *W*_0.1_ < 2 min in all three column conditions; however, in the P and P–S columns, the peak widths (*W*_0.1_) and their variability (standard deviations, SDs) tended to increase as the compounds retained more strongly (Figure 3b).

The first observation may be explained by the number of polar functional groups in the stationary phase. When glycerol dimethacrylate-based bare polymer particles were modified with epichlorohydrin (P → P–S), two hydroxyl group per modified group was exposed on the stationary phase surface. Furthermore, modification of the P–S polymer with polyethylenimine (P–S → P–S–A) results in two or more polar amino groups on the surface of the modified group. Therefore, the difference in the RTs of the P, P–S, and P–S–A columns for polar metabolites was consistent with the number of H_2_O molecules hydrated to the polar functional groups of the stationary phase, suggesting that this drives HILIC separation.

To explain the exception in the first observation (i.e., propylamine and His), we simulated the molecular/ionic distribution of the 52 polar metabolites at pH 9.8. The results showed that only propylamine, lysine (Lys), and arginine (Arg) could be in the positively charged molecular state at 72, 25, and 17%, respectively (see p*K*_a_ and p*K*_b_ in the Supporting Information, Table S-3). This suggests that propylamine has a high percentage of positive charge even at pH 9.8, and ion exclusion effects are at work in LC/MS/MS analysis using the P–S–A column with a positively charged stationary phase. Thus, the retention order for propylamine was P–S–A column < P column < P–S column (Figure 3a). The other exception, His, showed a broad peak shape in the P or P–S columns, except for the P–S–A column (Figure 3c). Without considering the symmetry of the peaks, the elution start time for His was calculated from RT and *W*_0.1_ data. Elution start times were 5.1, 8.1, and 8.7 min for the P, P–S, and P–S–A columns, respectively; when using the P or P–S columns, His eluted as a broad peak, which may have contributed to the exceptional behavior of RT indexed by the peak top.

The interpretation of the second and third observations could be explained by the ionic interactions in the AEX mode, as explained previously (see Optimization of LC conditions using a mixed amines polymer column). In the LC/MRM chromatograms with P or P–S columns, many anionic metabolites in group (iv) showed broad peak shapes (Figure 3b,c). In contrast, in the case of LC/MS/MS with the P–S–A column, the modification of the primary to quaternary amine gave a positive charge to the stationary phase, which may have contributed to elution with good retention and peak shape for all anionic metabolites via ionic interactions in the AEX mode (Figure 3a–c).

**Figure 4:**
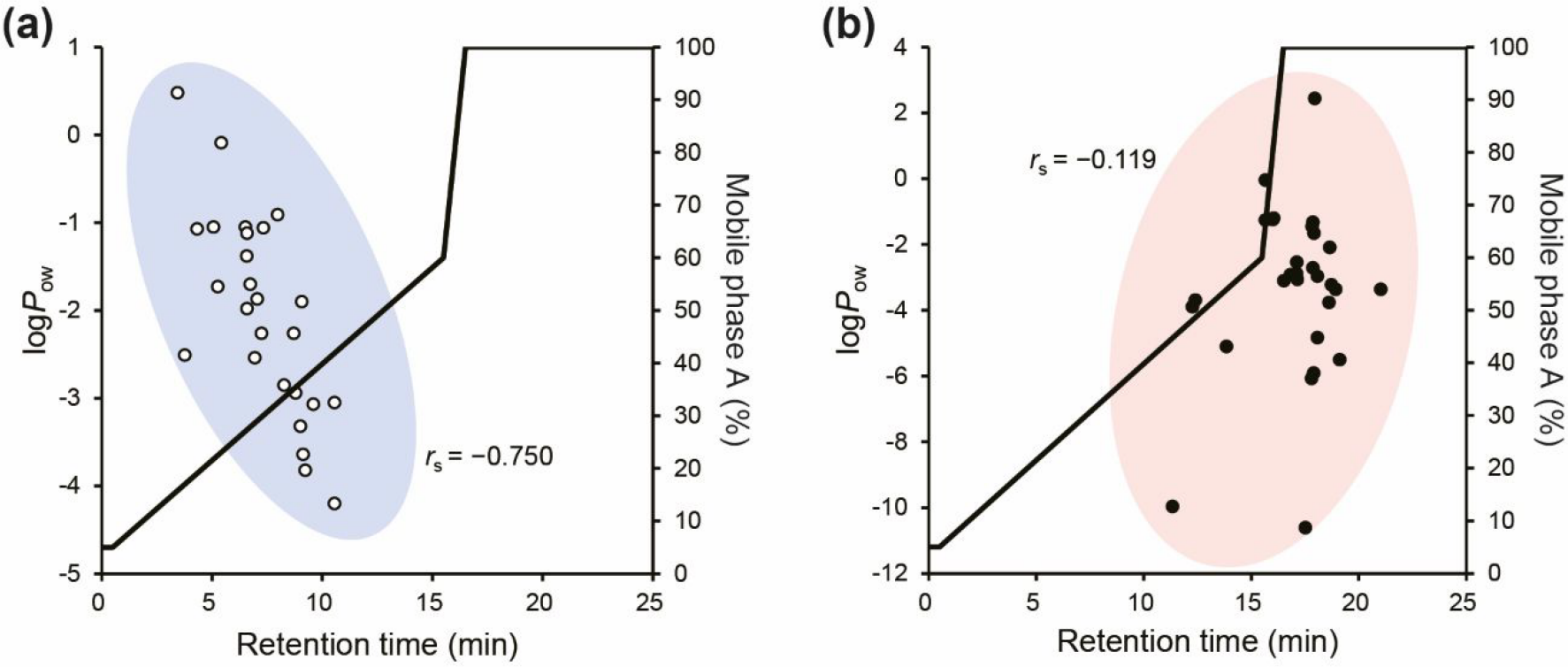
Relationship between RT and log *P*_ow_ for 52 polar metabolites in HILIC mode (a) and AEX mode (b) under optimized gradient elution conditions using a mixed amines polymer column. Mobile phase A, 40 mM ABC solution at pH 9.8; mobile phase B, acetonitrile. Other LC/MS/MS parameters are detailed in the Experimental Section.

The RT and log *P*_ow_ for each polar metabolite and aqueous mobile phase composition in the gradient method are described in Figure 4 to characterize the transition between the HILIC and AEX modes under optimized LC/MS/MS conditions (see the Experimental Section) using the mixed amines polymer column (P–S–A column). Spearman’s correlation analysis using RT and log *P*_ow_ values for each of the 52 polar metabolites showed that the correlation coefficient, *r*_s_, was −0.750 for cationic, uncharged, and zwitterionic polar metabolites in groups (i), (ii), and (iii) and −0.119 for hydrophilic metabolites in group (iv). HILIC is a chromatographic technique that uses more than 50% organic solvent in an aqueous–organic mobile phase with a polar stationary phase.^12^ For mobile phase compositions of 0–50% aqueous solvent with functioning HILIC mode, that is, RTs = 0–12.8 min, polar metabolites of groups (i), (ii), and (iii) were eluted, and there was a strong correlation between RT and log *P*_ow_ for these metabolites (|*r*_s_| > 0.70).

In contrast, the anionic metabolites of group (iv) showed stronger retention than the polar metabolites of groups (i), (ii), and (iii). Anionic metabolites other than aspartic acid (Asp), glutamic acid (Glu), and NAD were eluted with a mobile phase composition of 50–100% aqueous solvent, which is not the HILIC working solvent composition (RTs > 12.8 min). No correlation was observed between RT and log *P*_ow_ (|*r*_s_| < 0.20), and the order of the retention was consistent with the order of the anionic strength (AMP < ADP < ATP). The higher the HCO_3_^–^/CO_3_^2–^ ion concentration in the aqueous mobile phase, the faster the elution of anionic metabolites (Figure 1). These results indicate that the anionic metabolites of group (iv) were separated based on the AEX-dominant mode (RTs > 12.8 min). Asp, Glu, and NAD had a net negative charge but had positively charged moieties in their molecular structure, even at pH 9.8. Therefore, because of the ion exclusion effect of the positively charged moieties, these three anionic metabolites were considered less retained than other anionic metabolites.

To summarize the separation behavior of LC/MS/MS with the mixed amines polymer column, cationic, uncharged, and zwitterionic polar metabolites were separated based on the HILIC-dominant mode (RTs = 0–12.8 min), whereas anionic metabolites were adsorbed on the positively charged mixed amines stationary phase through ionic interactions. Subsequently, the adsorbed anionic metabolites eluted owing to increased HCO_3_^–^ or CO_3_^2–^ ions and were separated in the AEX-dominant mode (RTs = 12.8–26.5 min). A new single-run LC/MS method for the comprehensive analysis of polar metabolites, characterized by a continuous transition of separation mode from the HILIC to AEX mode, was termed “unified-HILIC/AEX/MS”.

### Comparative Evaluation of the Mixed Amines Polymer Column

To compare the chromatographic performance of the unified-HILIC/AEX, 52 selected polar metabolites were analyzed by LC/MS/MS using a bare-silica column, an amino-silica column, two types of zwitterionic-polymer columns (iHLIC-Fusion (P) or ZIC-pHILIC), and a mixed amines polymer column. The pH of the aqueous mobile phase used in the experiments was 7.0 for the silica-based columns, 7.0 and 9.8 for the zwitterionic-polymer column (iHLIC-Fusion (P)), and 9.8 for the mixed amines polymer column. Other LC and MS/MS (MRM) conditions were performed under the same conditions optimized in unified-HILIC/AEX/MS/MS. The RTs and peak widths (*W*_0.1_) obtained from LC/MS/MS under each condition are shown in Supporting Information, Figure S-5a,b and Table S-6. The LC/MS/MS chromatograms of His and ATP are shown in Supporting Information, Figure S-5c. The number of detected metabolites from each condition were different: bare-silica column (pH 7.0), 48 metabolites; amino-silica column (pH 7.0), 40 metabolites; zwitterionic-polymer column (iHLIC-Fusion (P); pH 7.0), 50 metabolites; and zwitterionic-polymer column (iHLIC-Fusion (P); pH 9.8) and mixed amines polymer column (pH 9.8), all 52 metabolites. Although all 52 polar metabolites were detected using a zwitterionic-polymer column (iHLIC-Fusion (P); pH 9.8), 14 of the 52 metabolites were eluted as a broad peak shape with *W*_0.1_ > 1 min (maximum *W*_0.1_, 3.6 min). Contrastingly, when a mixed amines polymer column (pH 9.8) was used, all 52 metabolites were eluted as a sharp peak with *W*_0.1_ < 1 min (maximum *W*_0.1_, 0.5 min; Supporting Information, Figure S-5b).

Because silane ligands on silica supports are hydrolyzed and removed by a high-pH mobile phase, an aqueous mobile phase, typically above pH 8.0, is not available in silica-based columns.^28^ Because CO_3_^2–^ ions, which are divalent anions, exist only under basic conditions above pH 9.0 (Supporting Information, Figure S-3a), silica-based columns could not utilize the AEX effects of CO_3_^2–^ ions. All metabolites that were not eluted with the amino-silica column (pH 7.0) were anionic in group (iv). Therefore, extending the available pH range to more than 9.0 using cationic or zwitterionic polymer columns would be effective in increasing the AEX effects for the elution of anionic metabolites. ZIC-pHILIC, a zwitterionic-polymer column, is most commonly used for the analysis of polar compounds.^29^ Two ZIC-pHILIC columns were prepared in this experiment; however, the back pressures of both columns increased after 10 analyses under the conditions optimized for the mixed amines polymer column, making it impossible to obtain data for comparison. A plausible reason for this might be the swelling/shrinking of the ZIC-pHILIC polymer particles upon rapidly changing the aqueous solution ratio from 100% to 5% of the initial solvent composition. Therefore, iHILIC-Fusion (P), also used for polar metabolites analysis,^17^ was used as a comparative polymer column because of its repeatability and stability. The RT and peak width (*W*_0.1_) results were sufficient to demonstrate the usefulness of the mixed amines polymer column for comprehensive polar metabolome analysis.

### Reassessment of Common Metabolomic Sample Preparation Methods Using Targeted Metabolome Analysis

By adding MRM and RT information for 400 hydrophilic metabolites, a targeted metabolome analysis method using unified-HILIC/AEX/MS/MS was developed. The results of the analytical validation are summarized in Supporting Information, Table S-2. The effect of sample solvent composition and injection volume on chromatographic peak shape was examined in Supporting Information, Figures S-6 and S-7. As long as the ratio of water to acetonitrile in the sample solvent is less than 50%, large sample volumes of up to 20 μL can be injected while maintaining peak shape and resolution. This feature is advantageous for high-sensitivity analysis with real samples.

The targeted metabolome analysis method was then used to investigate the effect of metabolomic sample preparation methods, that is, metabolite enrichment operations, on metabolomic data. CCFD is a commonly used technique to dry samples after Bligh and Dyer extraction to concentrate the metabolite extract or reconstitute the solvent composition.^30^ In contrast, the solvent composition of the upper hydrophilic layer after phase separation in Bligh and Dyer extraction was approximately water/methanol (4:5, v/v). Thus, the initial solvent for unified-HILIC/AEX was 2 mM ammonium bicarbonate in 95% acetonitrile, which allowed the samples extracted with organic solvent-rich solutions to be introduced directly into the analytical system without modifying the solvent composition through CCFD treatment. To determine the effect of CCFD treatment on the quantitative accuracy of polar metabolites, we compared the results of targeted metabolome analysis of HeLa cell extracts containing stable isotope-labeled standards (^18^O_9_-ATP and ^13^C_2_, ^15^N-GSH) from CCFD and DI samples (Supporting Information, Figure S-8). Targeted metabolomic analysis identified 160 polar metabolites and five labeled compounds (^18^O_9_-ATP, ^18^O_6_-ADP, ^18^O_3_-AMP, ^13^C_2_, ^15^N-GSH, and ^13^C_4_, ^15^N_2_-GSSG) (Supporting Information, Figure S-1 and Table S-7). To determine the stability of polar metabolites in the CCFD operations, the metabolomic profile data of the CCFD and DI samples were compared using a volcano plot (Supporting Information, Figure S-8a). Most polar metabolites were stable under CCFD treatment, whereas some hydrophilic metabolites increased or decreased, owing to their instability under CCFD treatment. Increases or decreases in several pairs of compounds, such as nucleoside triphosphates, nucleoside diphosphates, nucleoside monophosphates, *S*-adenosylmethionine (SAM), and *S*-adenosyl-l-homocysteine, were observed by the CCFD process (Supporting Information, Figure S-8a). ^18^O_9_-ATP spiked into HeLa cell extracts was significantly reduced by CCFD treatment, whereas ^18^O_6_-ADP and ^18^O_3_-AMP, the expected degradation products, were significantly increased (Supporting Information, Figure S-8b). This phenomenon was also observed when only the ^18^O_9_-ATP standard was used (Supporting Information, Figure S-8c). Some of the hydrophilic metabolites were affected by degradation (e.g., hydrolysis) in the CCFD process. Therefore, the unified-HILIC/AEX/MS/MS method, which allows the direct analysis of extracts, is expected to be a tool for obtaining accurate and comprehensive polar metabolome information.

### Evaluation of Polar Metabolite Coverage of the Unified-HILIC/AEX/HRMS Method Using Nontargeted Metabolomics

Since nontargeted analysis does not intentionally select analytes, the detected peak features can be used to evaluate the analyte coverage of the metabolome.^18^ To compare and evaluate the analytical coverage, we performed a nontargeted metabolomic analysis of HeLa cell extracts using the developed unified-HILIC/AEX/HRMS method utilizing the mixed amines polymer column (GL-HilicAex) and the conventional HILIC/HRMS method utilizing the zwitterionic-polymer column (iHILIC-Fusion (P)). Except for the column, the LC and HRMS conditions were identical for both methods. After background subtraction using a procedure blank sample, 3242 metabolic features were found using the unified-HILIC/AEX/HRMS method and 2068 using the conventional HILIC/HRMS method. Using the unified-HILIC/AEX/HRMS method, the number of metabolic features detected by single-run analysis increased by approximately 1.6-fold compared with that using the conventional HILIC/HRMS method. The unified-HILIC/AEX/MS analytical method is capable of separating a wide range of polar metabolites with different charge characteristics while maintaining sharp peak shapes (Figure 2). In the present study, the number of polar metabolites detected increased with improved chromatographic performance, allowing us to propose an innovative single-run polar metabolome analysis method. In the future, our single-run method for polar metabolomics is expected to be applied to cohort studies and analysis of small samples, such as autopsy tissue and single cells.^31^

## Conclusions

Herein, we proposed unified-HILIC/AEX/MS methods (unified-HILIC/AEX/MS/MS and unified-HILIC/AEX/HRMS) that can potentially achieve comprehensive and simultaneous measurement of the polar metabolome in a single run. The key factors in the unified-HILIC/AEX/MS methods are the use of a mixed amines polymer column for the stationary phase, carbonate ions generated at pH 9.8 at an appropriate concentration in the aqueous mobile phase, and a salt gradient with a final replacement of the acetonitrile-rich condition with a 100% 40 mM ammonium bicarbonate solution (pH 9.8). We found that cationic, uncharged, and zwitterionic polar compounds eluted with HILIC-dominant separation in the first half of the analysis, while anionic polar compounds eluted with AEX-dominant separation in the second half, enabling simultaneous analysis of the hydrophilic metabolome using a single-run method. The unified-HILIC/AEX/MS methods showed to better performance than conventional HILIC/MS methods. Unified-HILIC/AEX/MS/MS and unified-HILIC/AEX/HRMS were used for targeted and nontargeted metabolomic analyses, respectively, using HeLa cell extracts. The initial solvent for unified-HILIC/AEX was 2 mM ammonium bicarbonate in 95% acetonitrile, which allowed the samples extracted with organic solvent-rich solutions to be introduced directly into the analytical system. This characteristic allowed accurate quantitative information to be obtained even for unstable metabolites, such as ATP and SAM, which are partially degraded during drying operations by CCFD. Nontargeted metabolomics results demonstrated the effectiveness of our novel single-run analytical method, with improved metabolic features detected by unified-HILIC/AEX/HRMS compared to conventional HILIC-based methods. Overall, unified-HILIC/AEX/MS has the potential to become the first choice of an analytical tool for polar metabolome analysis.

## Supporting Information Available

(1) Cell culture; (2) Metabolomic sample preparation; (3) MRM conditions; (4) HRMS conditions; (5) Details of peak alignment and detection; Figures S-1–S-8 (PDF)
Tables S-1–S-7 (XLSX)
