# Supplementary material for: Unified-Hydrophilic-Interaction/Anion-Exchange Liquid Chromatography Mass Spectrometry (Unified-HILIC/AEX/MS): A Single-Run Method for Comprehensive and Simultaneous Analysis of Polar Metabolome
Source: Anal Chem. 2022 Nov 25;94(48):16877–86. doi: 10.1021/acs.analchem.2c03986 (PMC9730297; doi:10.1021/acs.analchem.2c03986)
Supplement: Supplementary file 1 — ac2c03986_si_001.pdf [file ac2c03986_si_001.pdf]

Supporting information

Unified-hydrophilic-interaction/anion-exchange liquid chromatography mass spectrometry (unified-HILIC/AEX/MS): A single-run method for comprehensive and simultaneous analysis of polar metabolome

Author names

Kohta Nakatani[†], Yoshihiro Izumi[*,†,‡], Masatomo Takahashi[†,‡], Takeshi Bamba[*,†,‡]

Author affiliations

[†]Division of Metabolomics/Mass Spectrometry Center, Medical Research Center for High Depth Omics, Medical Institute of Bioregulation, Kyushu University, 3-1-1 Maidashi, Higashi-ku, Fukuoka 812-8582, Japan

[‡]Department of Systems Life Sciences, Graduate School of Systems Life Sciences, Kyushu University, 3-1-1 Maidashi, Higashi-ku, Fukuoka 812-8582, Japan

*Corresponding authors

E-mail: izumi@bioreg.kyushu-u.ac.jp. Tel.: +81-92-642-6171.

E-mail: bamba@bioreg.kyushu-u.ac.jp. Tel.: +81-92-642-6170.

Contents

Cell culture

Metabolomic sample preparation.

MRM conditions.

HRMS conditions.

Details of peak alignment and detection.

Figure S-1. MRM transitions for unlabeled (ATP, ADP, AMP, GSH, and GSSG) and stable-isotope-labeled metabolites ($^{18}O_9$-ATP, $^{18}O_6$-ADP, $^{18}O_3$-AMP, $^{13}C_2$, ^{15}N-GSH, and $^{13}C_4$, $^{15}N_2$-GSSG).

Figure S-2. Workflow of DI and CCFD sample preparation methods.

Figure S-3. Molecular/ionic distributions of additives in the mobile phase (a), functional groups in stationary phase (b), and analyte (c) simulated using ChemAxon software.

Figure S-4. Comparison of retention behavior of zwitterionic and anionic compounds in aqueous mobile phases at pH 3.6, 7.0, and 9.8.

Figure S-5. Comparison of chromatographic performance of four different columns using RTs (a) and peak widths at 10% peak height ($W_{0.1}$) (b) for 52 polar metabolites. The columns and pH of the aqueous mobile phase are as follows: a bare-silica column (Inertsil SIL-100A), pH 7.0; an amino-silica column (Inertsil NH2), pH 7.0; a zwitterionic-polymer column (iHLIC-Fusion (P)), pH 7.0 and pH 9.8; and a mixed amines polymer column (GL-HilicAex), pH 9.8. Other LC and MS/MS conditions for all methods were identical. Values are presented as the mean ± standard deviation (n = 3). (c) LC/MS/MS chromatograms of His and ATP under five different conditions.

Figure S-6. Effect of sample solvent composition and injection volume on chromatographic peak shape. Leucine and isoleucine were dissolved in various ratio of water (W) and acetonitrile (A) solvent.

Figure S-7. Effect of sample solvent composition on chromatographic peak shape. Standard compounds were reconstituted with various composition of water (W), methanol (M), or acetonitrile (A).

Figure S-8. Effect of CCFD treatment on the accuracy of polar metabolite profiling. (a) Volcano plot showing individual HeLa cell metabolic profiles between CCFD and DI samples ($n = 3$). Red closed circles indicate statistically significant differences (*$P < 0.05$, **$P < 0.01$, and ***$P < 0.001$) using Student's *t*-tests. The relative abundance of $^{18}O_9$-ATP, $^{18}O_6$-ADP, and $^{18}O_3$-AMP in HeLa cell extracts or $^{18}O_9$-ATP standards added to HeLa cell extracts alone in DI or CCFD treatments. The relative abundance of $^{18}O_9$-ATP, $^{18}O_6$-ADP, and $^{18}O_3$-AMP on adding the $^{18}O_9$-ATP standard to HeLa cell extracts (b) or treating only the $^{18}O_9$-ATP standard (c) with DI or CCFD. Values are presented as the means ± standard deviations obtained from triplicate experiments.

Cell culture.

HeLa cells (American Type Culture Collection) were cultured in 10 cm diameter dishes containing 10 mL of DMEM supplemented with 10% (v/v) FBS and 1% (v/v) penicillin-streptomycin solution for antibiotics. Cultivation dishes were incubated in a water-jacketed CO_2 incubator (WCI-165; ASTEC Co., Fukuoka, Japan) under an atmosphere of 5% CO_2 at 37 °C. When cells were cultured to 80% confluence, the medium in each dish was changed 1 h before cell sampling ($n = 6$). Trypsin-EDTA-treated HeLa cells ($n = 1$) were counted using a cell counter (Moxi Z; ASONE Co., Osaka, Japan).

Metabolomic sample preparation.

After removing the culture medium (~10 mL) by aspiration, HeLa cells were washed three times with 10 mL of cold PBS (4 °C) and quenched with 1 mL of cold methanol (−30 °C) on ice. After scraping, the cell suspension (approximately 3.9×10^6 cells) ($n = 5$) was transferred into a 2 mL Eppendorf tube and mixed with 400 μL of chloroform and 5 μL of a stable isotope-labeled standard solution containing $^{18}O_9$-ATP (2.5 nmol) and $^{13}C_2$, ^{15}N-GSH (2.5 nmol). The samples were vigorously mixed by vortexing for 1 min, followed by sonication for 5 min. After centrifugation for 5 min at 16 000*g* at 4 °C, 700 μL of the supernatant was transferred to a clean tube. Subsequently, 300 μL of chloroform and 400 μL of water were added and vortexed. After centrifugation for 3 min at 16 000*g* at 4 °C, the aqueous (upper) layer (400 μL) was divided into 200 μL for direct injection (DI) samples and 200 μL for centrifugal concentration/freeze-drying (CCFD) samples. The CCFD samples were removed from the methanol in a centrifugal evaporator for approximately 20 min, frozen in liquid nitrogen, and lyophilized overnight. The CCFD samples were dissolved in 200 μL water/methanol (4:5, v/v). The DI and CCFD solutions were transferred into LC vials and stored at −80 °C until LC/MS/MS or LC/HRMS analysis.

MRM conditions.

The MRM mode was applied to all targeted LC/MS/MS analyses. The MS conditions were as follows: nebulizer gas flow, 2 L/min; heating gas flow, 10 L/min; drying gas flow, 10 L/min; heat block temperature, 400 °C; desolvation line temperature, 250 °C; and spray voltage, 4.0 kV for positive ion mode and −3.0 kV for negative ion mode. The MRM parameters were as follows: dwell time, 2 ms; pause time, 2 ms; and polarity switching time, 5 ms. The other MRM conditions,

including the Q1 pre-bias, collision energy, and Q3 pre-bias of each metabolite, were automatically optimized by flow injection analysis with standard solutions (1−100 μM) using LabSolution ver. 5.91 (Shimadzu Co.).

HRMS conditions.

The full scanning HRMS analysis conditions were as follows: polarity, positive and negative ionization; sheath gas flow rate, 40 arb; auxiliary (Aux) gas flow rate, 10 arb; spray voltage, 3.5 kV for positive ion mode and −2.5 kV for negative ion mode; capillary temperature, 275 °C; S-lens level, 50; heater temperature, 425 °C; mass resolution, 70 000; automatic gain control (AGC) target (the number of ions to fill C-Trap), 1×10^6; maximum injection time, 200 ms; and scan range, 100−700 (*m/z*).

Details of peak alignment and detection.

Compound Discoverer ver. 3.0 (Thermo Fisher Scientific Inc.) was used for data processing, including peak alignment (node name: align retention times), peak detection (node name: detect unknown compounds), data grouping (node names: group unknown compounds and fill gaps), and background subtraction (node name: mark background compounds). The parameters for the align retention times are follows: alignment model, adaptive curve; mass tolerance, 10 ppm; and maximum retention shift, 0.1 min. The extracted ion traces for LC/HRMS full-scan data were detected using the parameter settings for the mass tolerance, intensity threshold, and isotopic pattern. The same chemical features derived from isotopes and adducts were combined with the most abundant peak using a user-specified ions list and RT information. The peak area was then calculated for each compound. The optimized automatic filter criteria to detect unknown compounds (peak detection, grouping of isotopes and adducts, and calculation of peak area) were as follows: mass tolerance, 10 ppm; intensity tolerance for isotopic pattern search, 50%; *S/N* threshold, 3; minimum peak intensity, 10 000; adducts ions, $[M+H]^+$, $[M+Na]^+$, $[M+NH_4]^+$, $[M-H]^-$, and $[M-H-H_2O]^-$; minimum element counts, CHNOPS; maximum element counts, C90H190N10O18P3S5; filter peaks, true; maximum peak width, 0.6 min; remove singlets, true; minimum scans per peak, 3; and minimum isotopes, 1. The following parameters for the group unknown compound were used to construct a data matrix consisting of the RT, exact mass, and peak area across the sample set: mass tolerance, 10 ppm; and RT tolerance, 0.1 min. The parameters

for the fill gap were as follows: mass tolerance, 10 ppm; and *S/N* threshold, 10. The parameters for the background-subtraction process (i.e., mark background compound) between procedure blank and HeLa cells samples were as follows: maximum peak area (sample/blank), 2; and hide background; true.

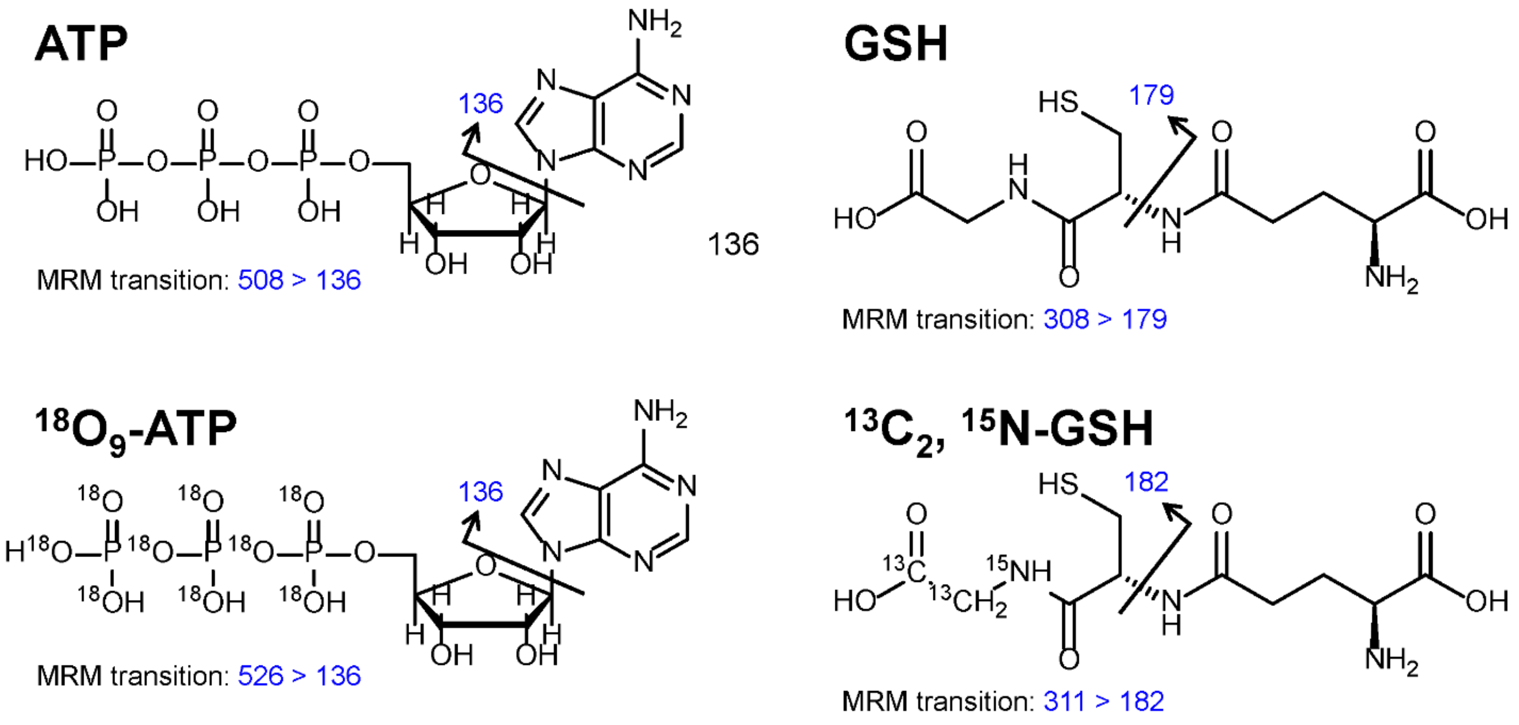

ADP MRM transition: 428 > 136

$^{18}O_6$-ADP MRM transition: 440 > 136

AMP MRM transition: 348 > 136

$^{18}O_3$-AMP MRM transition: 354 > 136

GSSG MRM transition: 613 > 355

$^{13}C_4$, $^{15}N_2$-GSSG MRM transition: 619 > 361

Figure S-1. MRM transitions for unlabeled (ATP, ADP, AMP, GSH, and GSSG) and stable-isotope-labeled metabolites ($^{18}O_9$-ATP, $^{18}O_6$-ADP, $^{18}O_3$-AMP, $^{13}C_2$, ^{15}N-GSH, and $^{13}C_4$, $^{15}N_2$-GSSG).

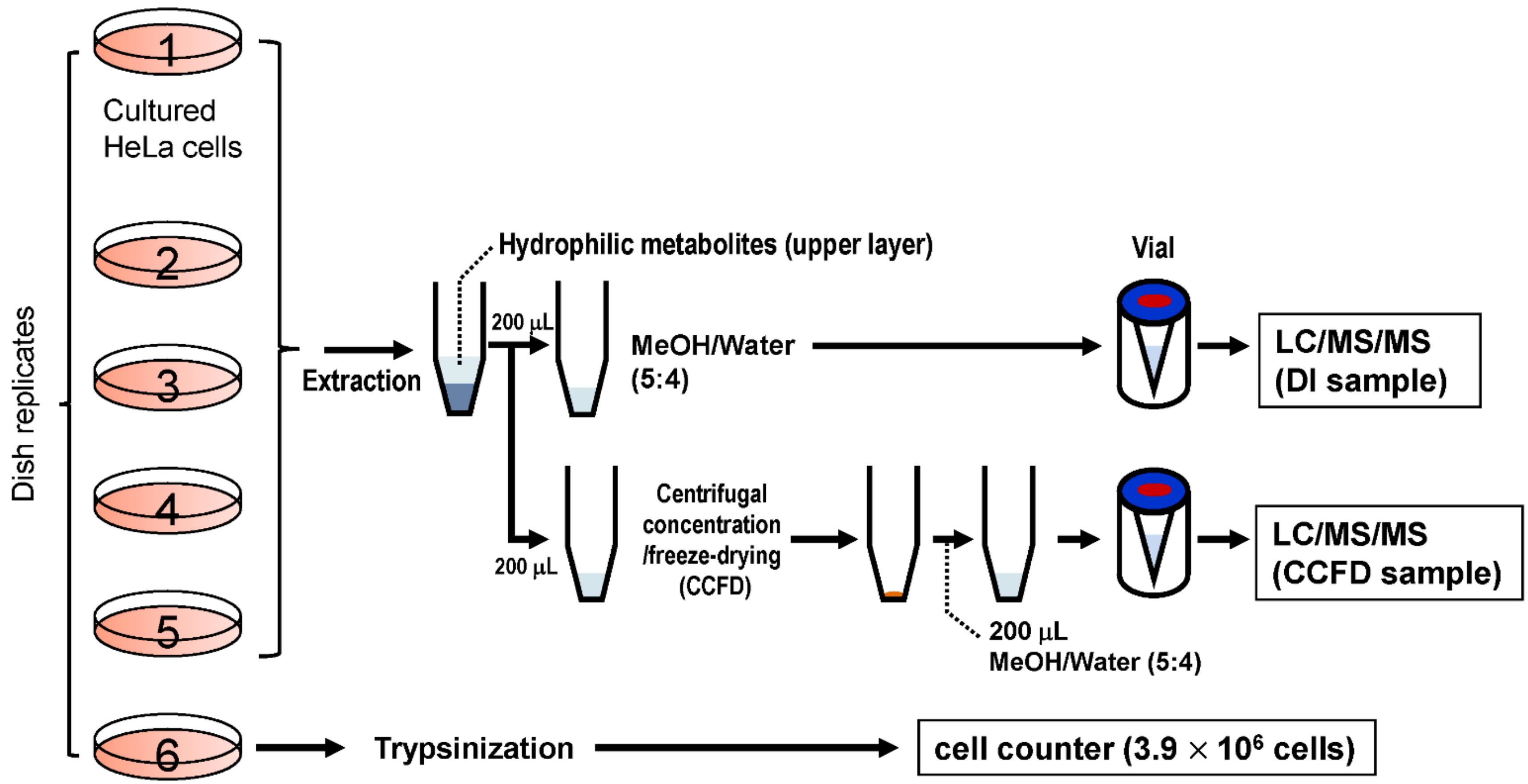

Figure S-2. Workflow of DI and CCFD sample preparation methods.

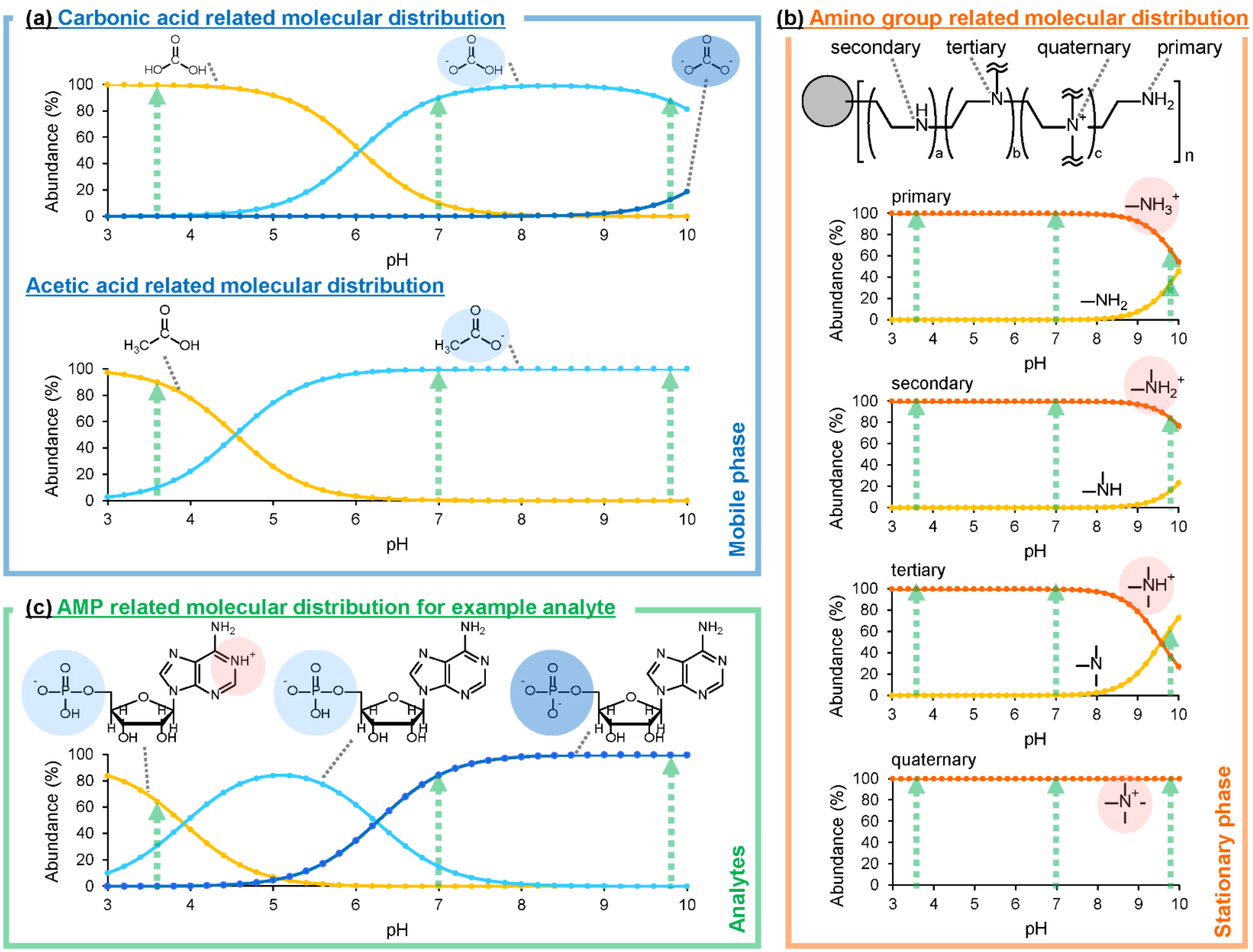

Figure S-3. Molecular/ionic distributions of additives in the mobile phase (a), functional groups in stationary phase (b), and analyte (c) simulated using ChemAxon software.

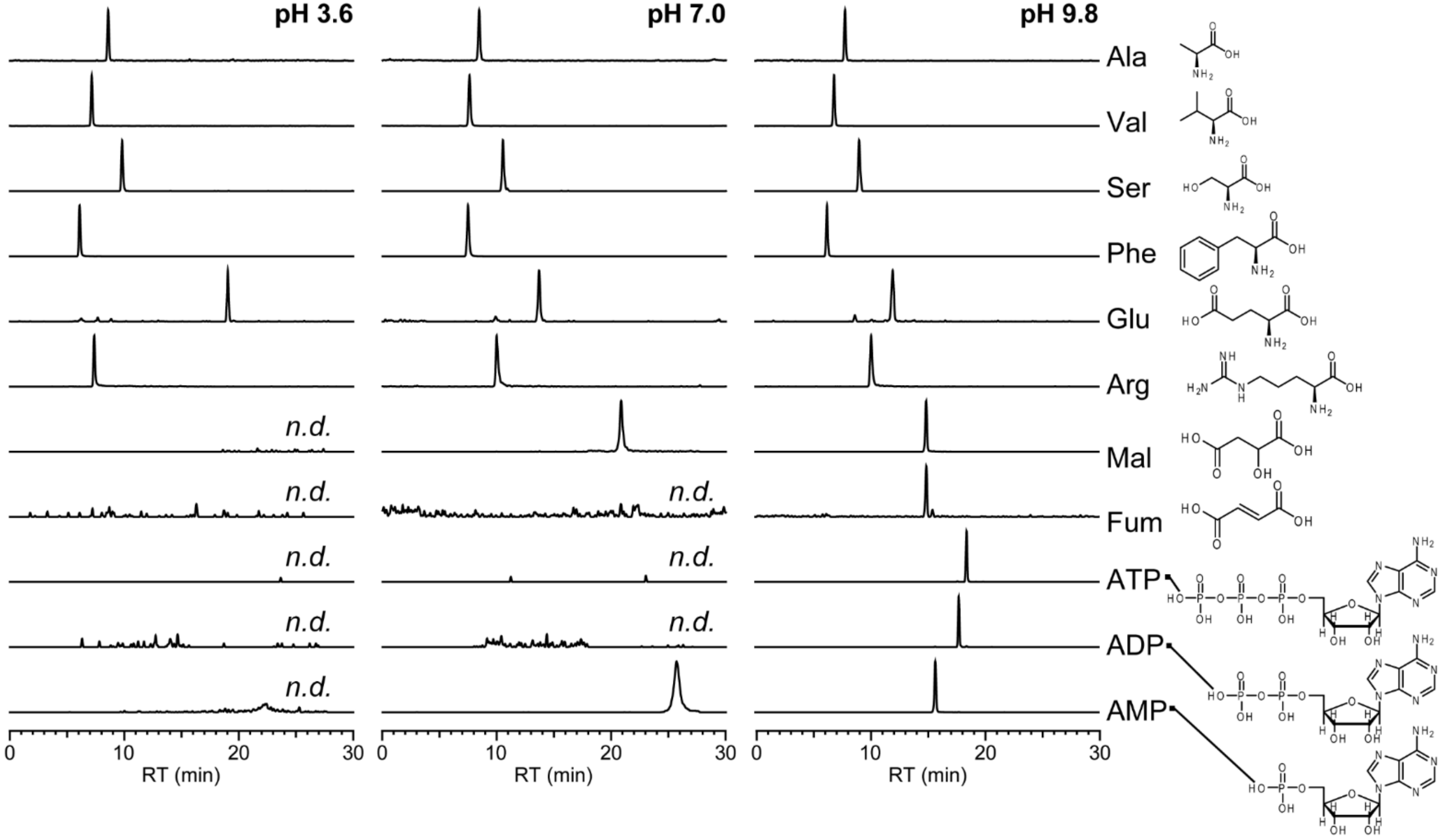

Figure S-4. Comparison of retention behavior of zwitterionic and anionic compounds in aqueous mobile phases at pH 3.6, 7.0, and 9.8.

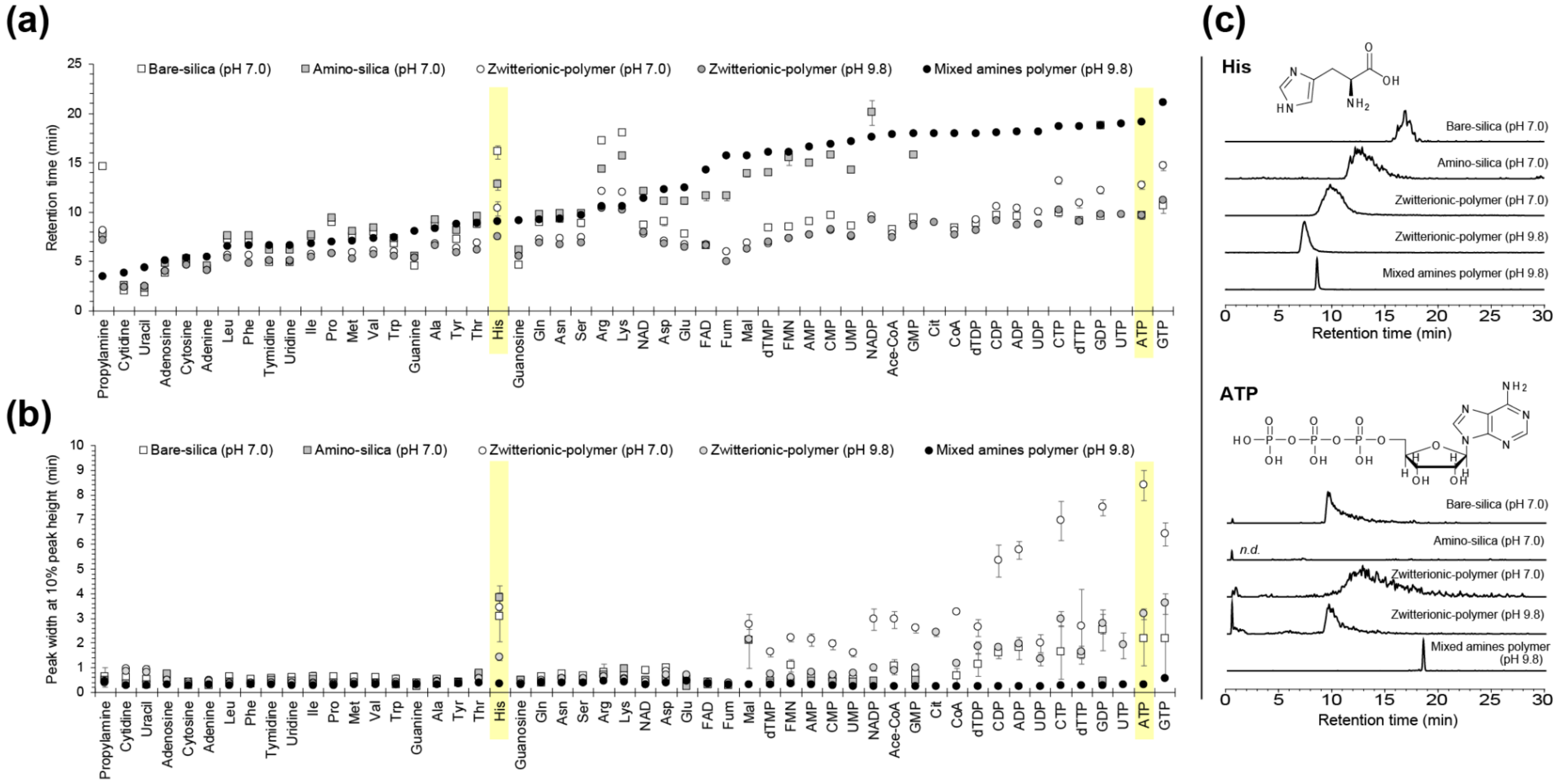

Figure S-5. Comparison of chromatographic performance of four different columns using RTs (a) and peak widths at 10% peak height ($W_{0.1}$) (b) for 52 polar metabolites. The columns and pH of the aqueous mobile phase are as follows: a bare-silica column (Inertsil SIL-100A), pH 7.0; an amino-silica column (Inertsil NH2), pH 7.0; a zwitterionic-polymer column (iHLIC-Fusion (P)), pH 7.0 and pH 9.8; and a mixed amines polymer column (GL-HilicAex), pH 9.8. Other LC and MS/MS conditions for all methods were identical. Values are presented as the mean ± standard deviation (*n* = 3). (c) LC/MS/MS chromatograms of His and ATP under five different conditions.

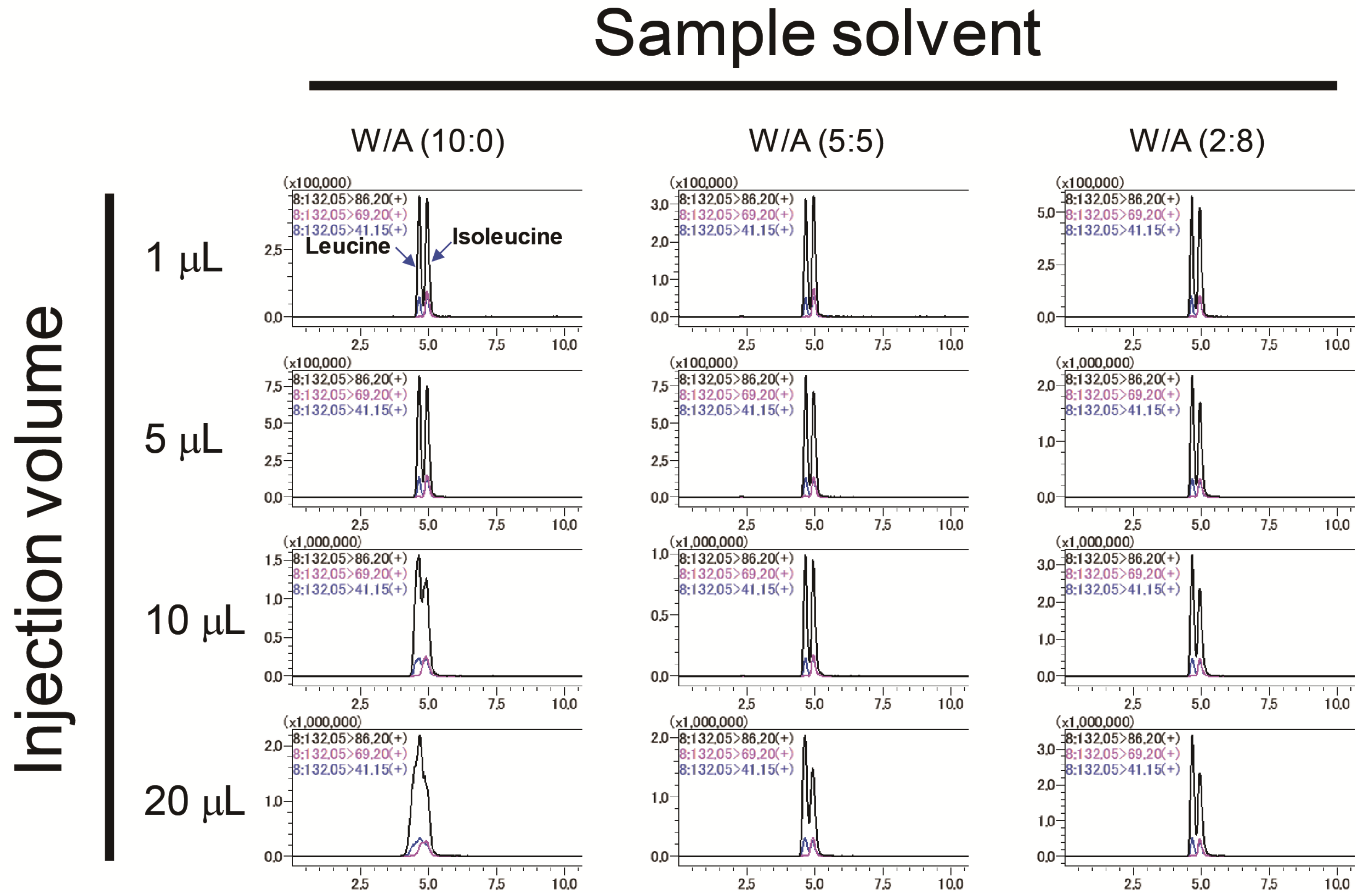

Figure S-6. Effect of sample solvent composition and injection volume on chromatographic peak shape. Leucine and isoleucine were dissolved in various ratio of water (W) and acetonitrile (A) solvent.

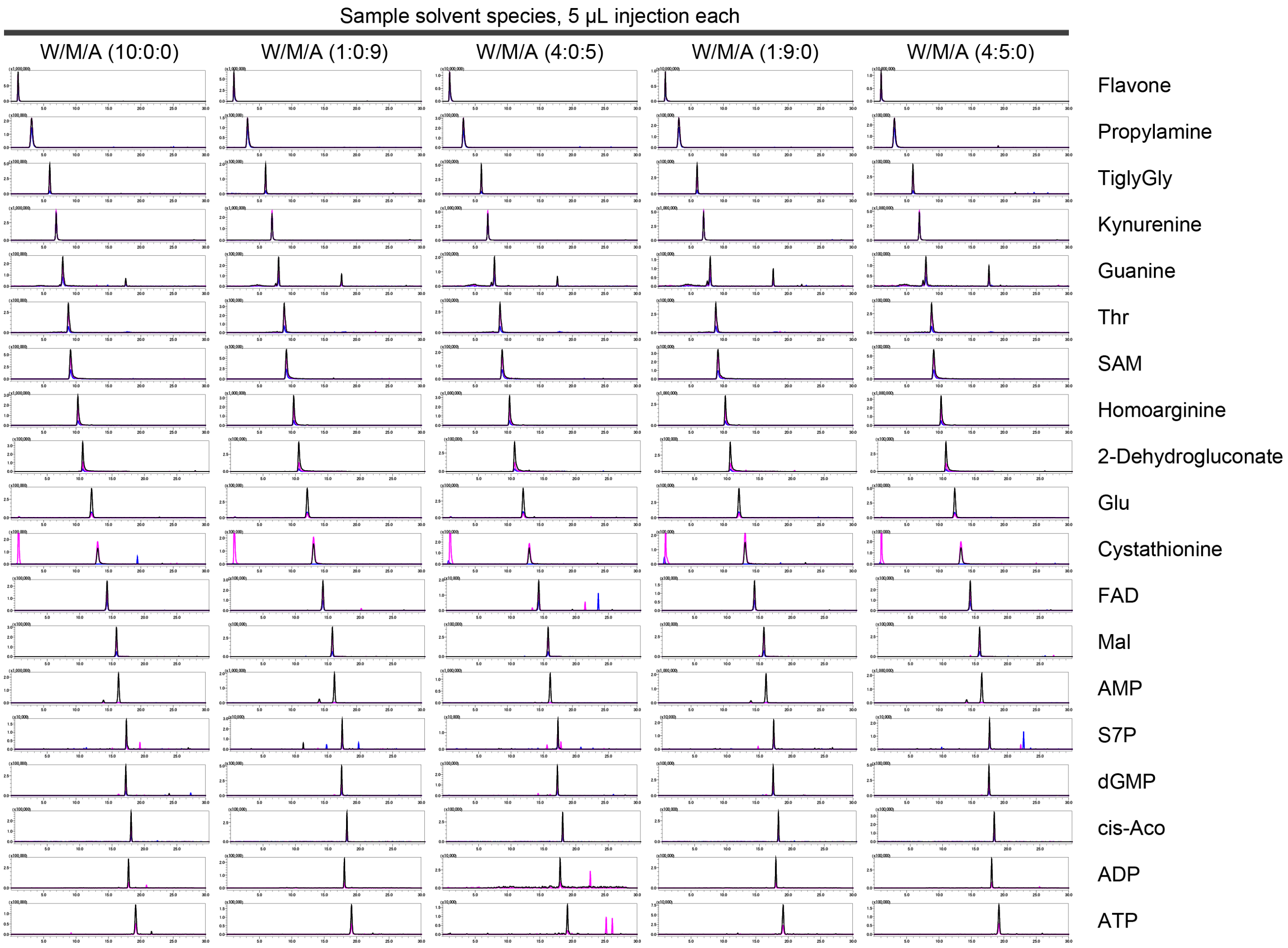

Figure S-7. Effect of sample solvent composition on chromatographic peak shape. Standard compounds were reconstituted with various composition of water (W), methanol (M), or acetonitrile (A).

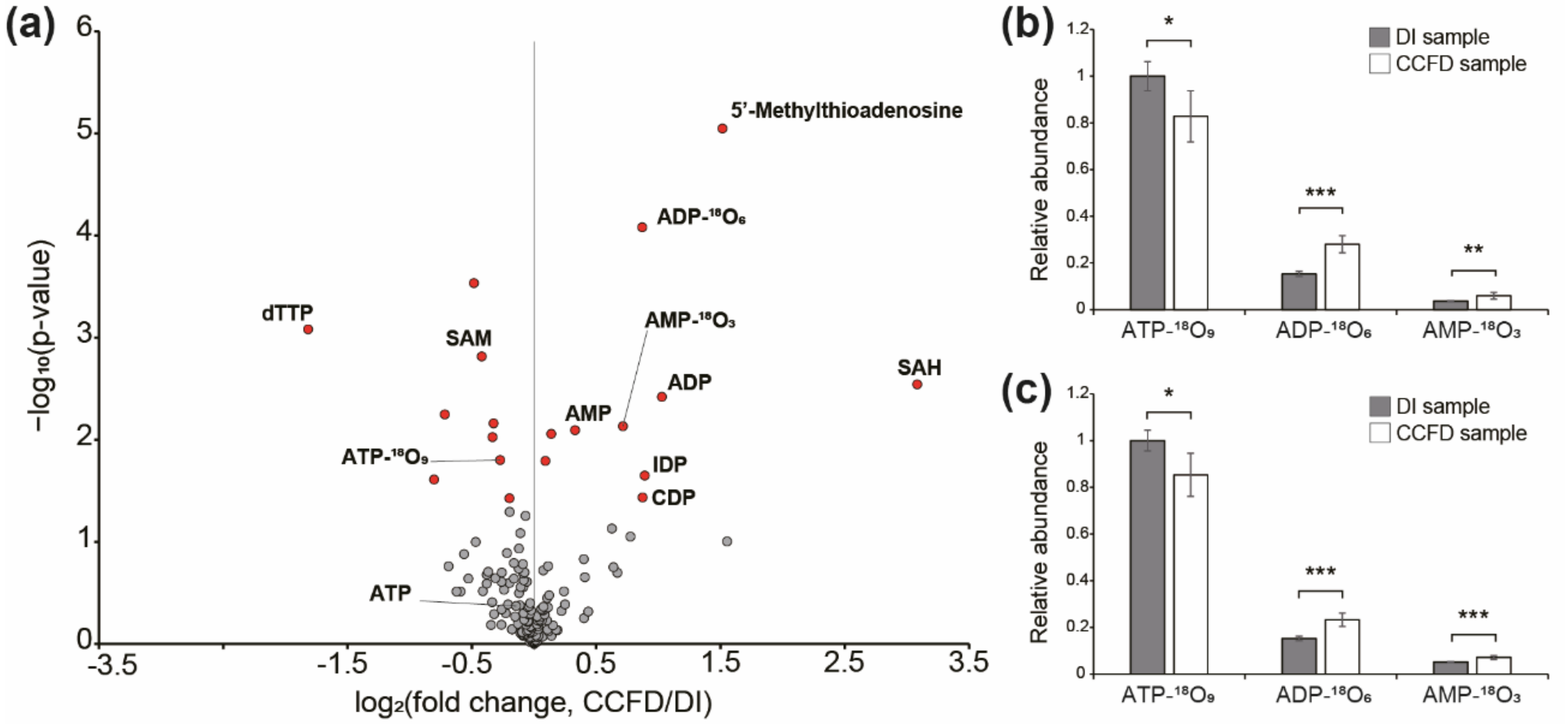

Figure S-8. Effect of CCFD treatment on the accuracy of polar metabolite profiling. (a) Volcano plot showing individual HeLa cell metabolic profiles between CCFD and DI samples (n = 3). Red closed circles indicate statistically significant differences (*$P < 0.05$, **$P < 0.01$, and ***$P < 0.001$) using Student's t-tests. The relative abundance of $^{18}O_9$-ATP, $^{18}O_6$-ADP, and $^{18}O_3$-AMP in HeLa cell extracts or $^{18}O_9$-ATP standards added to HeLa cell extracts alone in DI or CCFD treatments. The relative abundance of $^{18}O_9$-ATP, $^{18}O_6$-ADP, and $^{18}O_3$-AMP on adding the $^{18}O_9$-ATP standard to HeLa cell extracts (b) or treating only the $^{18}O_9$-ATP standard (c) with DI or CCFD. Values are presented as the means ± standard deviations obtained from triplicate experiments.
